# Probability and amount of medicines expenditure according to health insurance status in Kenya: A household survey in eight counties

**DOI:** 10.1002/hpm.3368

**Published:** 2021-10-21

**Authors:** Veronika J. Wirtz, Edson Servan‐Mori, John Mungai, John Mboya, Peter C. Rockers, Monica A. Onyango, Zana Wangari Kiragu, Richard Laing

**Affiliations:** ^1^ Department of Global Health Boston University School of Public Health Boston Massachusetts USA; ^2^ Center for Health Systems Research National Institute of Public Health Cuernavaca Mexico; ^3^ Innovation for Poverty Action Nairobi Kenya; ^4^ School of Public Health University of Western Cape Cape Town South Africa

**Keywords:** health insurance, Kenya, medicines expenditure, universal health coverage

## Abstract

**Background:**

National and county governments in Kenya have introduced various health insurance schemes to protect households against financial hardship as a result of large health expenditure. This study examines the relationship between health insurance and medicine expenditure in eight counties in Kenya.

**Methods:**

A cross‐sectional study of collected primary data via household survey in eight counties was performed. Three measures of medicine expenditure were analysed: the probability of any out‐of‐pocket expenditure (OOPE) on medicines in the last 4 weeks; amount of OOPE on medicines; and OOPE on medicines as a proportion of total OOPE on health.

**Results:**

Out of the 452 individuals, those with health insurance (*n* = 225) were significantly different from individuals without health insurance (*n* = 227): overall, they were older, had a higher level of educational attainment and possessed more assets. Adjusting for covariates, individuals with health insurance had a reduced probability of OOPE on medicines (0.40, CI95% 0.197–0.827) and spent proportionally less on medicines out of total health expenditure (0.50, CI95% 0.301–0.926).

**Conclusions:**

Kenya has made great strides to scale up Universal Health Coverage including access to medicines. Prioritising enrollment of low‐income individuals with non‐communicable diseases can accelerate access to medicines and financial protection.

## 1 INTRODUCTION

In many low‐ and middle‐income countries **(LMICs)** medicines are largely financed as out‐of‐pocket expenditure (OOPE) causing financial hardship and impoverishment.^1^ OOPE is the most inequitable financing mechanism since acquisition of goods depend on household wealth. Over the past decades the movement towards universal health coverage (UHC), now a core element of the Sustainable Development Goals (SDGs‐3.8), promote the implementation of public financing mechanisms to increase equitable access to health services including medicines and protect households from financial hardship.^2^ Many countries have prioritised roll‐out of health insurance to the poorest income groups of the population because of the greater need of financial protection in these households.^3^

Kenya has introduced different health insurances to protect households against financial hardship as a result of large health expenditure.^4^ The National Hospital Insurance Fund (NHIF) was introduced in 1960s as an insurance for those in formal employment which was later expanded to those working in the informal sector.^5^ Over the past decades, the NHIF has undergone many reforms and is currently the largest insurance fund in terms of beneficiaries across Kenya.^6^ Apart from the NHIF, Kenya has other large healthcare purchasers such as the national government, the county governments, and private insurers.^6^ Four counties, including Kisumu, Machakos, Nyeri and Isiolo, were pilot counties for the roll‐out of the nationwide **UHC** from December 2018 to March 2020.^7^, ^8^ Some counties have their own version of UHC such as Makueni.^9^

All of the healthcare purchasers, NHIF, government and county, offer a core benefit package. Even though there are some differences between the benefit packages of each healthcare purchaser, all of the packages include the non‐communicable disease (NCD) medicines that are part of the Kenya Essential Medicines List.^10^ It is expected that these three insurance schemes lower the probability of spending as well as the amount of spending on NCD medicines.

There are still gaps in our understanding about the effectiveness of the service coverage including medicines and financial protection of health insurances in Kenya. An analysis of the Kenya Household Health Expenditure and Utilisation Survey (KHHEUS) 2018 shows that poorer households in rural areas were particularly vulnerable to catastrophic payments.^11^ It also showed that having one or more household member suffering from chronic diseases was positively associated with the probability of incurring catastrophic expenditure.^11^ Earlier studies from 2003 and 2007 KHHEUS confirm that poorer households are more vulnerable to catastrophic payments, especially the second poorest and middle quintile.^12^ Depending on the methodology used, an estimated 1.5–2.5 million Kenyans being pushed below the national poverty line.^12^, ^13^ These studies did not focus on the role of medicines related expenditure and their contribution to catastrophic health expenditures. The studies also make a case for studying the causes of OOP in more detail and identifying effective financial protection schemes.^11^ Given the progress that Kenya has made over the past years in scaling up UHC it is important to study the extent to which the role‐out of UHC and county insurance schemes have been able to mitigate large payments related to health including medicines.

The aims of this study were twofold: to study the probability and amount of expenditure on medicines according to health insurance status of households and to analyse influence of health insurance on the probability and amount of expenditure on medicines by NCD patients.

## 2 METHODS

### 2.1 Setting/design and study participants

A cross‐sectional study of collected primary data was performed. The study formed part of a larger evaluation of a pricing scheme to enhance availability and affordability of NCD medicines at households of patients with NCDs called *Novartis Access*. The evaluation was conducted in eight counties in Kenya from 2016 to 2019. Data were collected at baseline, midline, and end line. The main purpose was to evaluate the impact of *Novartis Access* on the availability and price of NCD medicines included in the program portfolio and their therapeutic equivalents.^14^ The results of the evaluation are reported elsewhere.^15^ This study is using end line household survey data.

The selection of the eight study counties is described in detailed elsewhere.^14^ Briefly, counties were selected based on their security to enable a visit to the households for data collection and the county medicines purchasing status with Mission for Essential Drugs and Supplies, an organisation that supplies the medicines to not‐for‐profit public and private (often‐faith‐based) health facilities.

Ten study households in each of the eight counties were randomly selected from 10 administrative randomly selected enumeration areas, the smallest unit, equivalent to a village. Eligibility criteria of households were patients 18 years or older, previously diagnosed and prescribed medicine for diabetes, asthma, hypertension, dyslipidemia, or breast cancer. If the households had multiple members meeting the inclusion criteria, they were also invited to participate. The end line household survey asked about the household characteristics, assets, expenditures over the last year, medical related expenditures on health and medicines in the last four weeks as well as whether the household members had any form of health insurance (e.g., NHIF, government, county insurance).

### 2.2 Variables

#### 2.2.1 Out‐of‐pocket expenditure

We analysed three outcome variables: the probability of OOPE on medicines, the proportion of total medicines OOPE out of total OOPE on health ranged between zero to one, and OOPE amount spent on medicines. All expenditures were expressed in Kenyan Shillings (KES). The total OOPE on health was calculated by taking the sum of all expenditures reported by the individual in the last month before the survey, including registration/card, medicines/vaccines (including outside purchase), consultation, diagnosis tests (x‐ray, lab, etc.), medical check‐up, staying overnight in a hospital or health facility, and other expenditures. The unit of analysis is OOPE per individual.

#### 2.2.2 Health insurance

Our main exposure variable was the affiliation to any health insurance (e.g., NHIF, UHC, county) operationalised as a binary variable at the individual level (yes = 1, no = 0).

#### 2.2.3 Covariates

We recorded data on the sociodemographic and health condition characteristics of the surveyed participants. These were: age (years), sex (female = 1, male = 0), marital status (single, married or living together, divorced, separated or widowed), schooling level (preschool on none, not completed primary school, completed primary school, secondary school and higher), diagnosis of asthma, type 2 diabetes, or hypertension, and following previous studies^16^ an index based on the ownership of assets and housing infrastructure, which we constructed through factorial analysis using principal‐component factors.^17^ The viability and relevance of the index were confirmed through a correlation matrix, a determinant correlation matrix (0.05) and testing based on Bartlett's sphericity (*p* < 0.001) and the Kaiser–Meyer–Olkin (KMO = 0.75) techniques. Reliability was calculated using the α‐Cronbach (0.92).^18^ We broke down the index into terciles (low, middle and high socioeconomic levels).

#### 2.2.4 Analysis

All analyses were performed using the statistical package Stata MP v15.1.^19^ First, we described the sociodemographic and health differences of the studied participants according to insurance affiliation. We used bivariate regression models to compare both groups. We reported mean, percentage and CI95%. We then described the three outcome variables according to the health insurance.

The influence of health insurance on OOPE variables was analysed by estimating three regression models: First, for the probability of spending on medicines, we fit a multiple logistic regression model adjusted by the covariates mentioned above. Second, for the proportion of total medicines OOPE out of total OOPE on health, we estimate a fractional logistics multiple regression model.^20^, ^21^ These models estimate the parameters of interest using the quasi‐maximum likelihood method, adjusting the following log‐likelihood function:

$$\ln\;L=\overset{N}{\underset{j=1}{\sum }}y_{j}\ln\left\{G\left(x\prime_{j}\beta \right)\right\}+\left(1-y_{j}\right)\ln\left\{1-G\left(x\prime_{j}\beta \right)\right\}$$

where *N* is the sample size, $y_{j}$ the outcome variable; and G(.) the cumulative distribution function. This model was estimated using the *fracreg* command of the statistical package Stata MP v15.1.^19^ Adjusted Odds‐ratios and CI95% were reported.

Third, following previous studies,^22^, ^23^ and considering that health spending is not normally distributed, we used quantile regression to identify associations between health insurance and the amount of OOPE on medicines (among individuals with OOPE on medicines >0). We used quantile regression to be able to differentiate marginal changes in the OOPE medicines along the expenditure distribution in our sample. This method provides a richer characterisation of the data, allowing us to identify differential effects of covariates across our distribution, not merely its conditional mean.^24^, ^25^, ^26^ Our basic model form was:

$$\text{OOPE}=Q\left(\beta_{q}\text{NHIF},\gamma_{q}X\right)$$

where β is the quantile effect of NHIF on quantile *q*th of OOPE on medicines (percentile 50 in our case) and γ is a vector of the quantile (*q*th) effect of a vector *X* that includes adjusted covariates. This model was estimated using the *qreg* command of the statistical package.^19^ Coefficients and CI95% were reported.

## 3 RESULTS

Four hundred and fifty two individuals who reported any expenditure data on medicines in the last four weeks were included in the analysis (Table 1). Out of these 452 individuals, 225 reported not having any form of health insurance compared with 227 individuals reporting having some form of insurance. The individuals with health insurance were significantly different from individuals without health insurance: overall, they were older, a higher proportion were married or living together, had a higher level of educational attainment and possessed more assets. On one hand, more individuals diagnosed with asthma reported not having health insurance compared to the individuals with health insurance. On the other hand, more individuals diagnosed with diabetes reporting having health insurance compared to the individuals without health insurance.

**TABLE 1:** Sample characteristics

	Total (*n* = 452)	Without health insurance (*n* = 225, 49.8%)	With health insurance (*n* = 227, 50.2%)
	Mean, % and [CI95]		
Age (years)***	61.2 [59.6–62.7]	58.1 [55.8–60.5]	64.2 [62.3–66.1]
Female***	75.4 [71.5–79.4]	81.8 [76.7–86.8]	69.2 [63.1–75.2]
Marital status			
Single	4.4 [2.5–6.3]	5.3 [2.4–8.3]	3.5 [1.1–5.9]
Married or living together***	62.4 [57.9–66.9]	53.3 [46.8–59.9]	71.4 [65.5–77.3]
Divorced, separated, or widowed*	33.2 [28.8–37.5]	41.3 [34.9–47.8]	25.1 [19.4–30.8]
Schooling			
Preschool on none*	26.3 [22.3–30.4]	38.2 [31.8–44.6]	14.5 [9.9–19.1]
Primary school (not completed)	27.7 [23.5–31.8]	27.6 [21.7–33.4]	27.8 [21.9–33.6]
Primary school*	21.5 [17.7–25.3]	18.2 [13.2–23.3]	24.7 [19.0–30.3]
Secondary school**	16.2 [12.7–19.6]	12.4 [8.1–16.8]	19.8 [14.6–25.0]
Higher than secondary school***	8.4 [5.8–11.0]	3.6 [1.1–6.0]	13.2 [8.8–17.6]
Asset and housing index			
Low***	33.4 [29.0–37.8]	43.6 [37.0–50.1]	23.3 [17.8–28.9]
Middle*	33.4 [29.0–37.8]	37.8 [31.4–44.1]	29.1 [23.1–35.0]
High***	33.2 [28.8–37.5]	18.7 [13.6–23.8]	47.6 [41.0–54.1]
Had diseases			
Asthma***	22.6 [18.7–26.4]	31.1 [25.0–37.2]	14.1 [9.5–18.6]
Diabetes***	23.9 [19.9–27.8]	16.9 [12.0–21.8]	30.8 [24.8–36.9]
Hypertension***	74.8 [70.8–78.8]	68.9 [62.8–75.0]	80.6 [75.4–85.8]

***p < 0.01, **p < 0.05, *p < 0.10.

The overall probability of OOPE on health in the last four weeks was 41% with no significant difference between those with and without health insurance (Table 2). The amount of health expenditure in the last 4 weeks was similar for both groups (1340 KES which is equivalent to $US 13 dollars). There was a statistically significant difference in the probability of medicines expenditure and the proportion of medicine expenditure out of the total health expenditure between individuals having health insurance and those without. Individuals with health insurance had a lower probability of OOPE than those without and their proportion spent on medicines out of total health expenditure was lower than those without insurance.

**TABLE 2:** Overall probability and the amount of out‐of‐pocket expenditure on health and medicines

	Total	Without health insurance	With health insurance
	Mean, % and [CI95]		
Probability of OOPE in health, %	41.4 [36.8–45.9]	38.2 [31.8–44.6]	44.5 [38.0–51.0]
OOPE in health (in the last month, KES), p50 and IQR	1340 [600–3950]	1370 [600–4220]	1280 [600–3800]
Probability of OOPE in medicines**	61.5 [54.5–68.5]	69.8 [59.9–79.6]	54.5 [44.6–64.3]
OOPE in drugs/OOPE in health, %**	67.7 [62.3–73.0]	49.3 [40.7–57.8]	35.1 [24.7–42.8]
OOPE in medicines (in the last month, KES), p50 and IQR	1066.7 [540–2400]	961.0 [435–2253.6]	1100 [718.6–2550]

Abbreviations: IQR, interquartile range; KES, Kenyan Shillings; OOPE, out‐of‐pocket expenditure; p50 median.

**p < 0.05.

The statistical model confirms the descriptive results: individuals with health insurance have a reduced probability of OOPE on medicines (0.40, CI95% 0.197–0.827), spent proportionally less on medicines out of total health expenditure (0.50, CI95% 0.301–0.926) (Table 3). There was no difference in the amount spend on medicines between the two groups.

**TABLE 3:** Influence of health insurance on out‐of‐pocket expenditure on medicines

	Probability of OOPE in medicines	OOPE in drugs/OOPE in health	OOPE in medicines (KES, p50)
	aOR and CI95%	aOR and CI95%	Coeff. and CI95%
Health insurance	0.40**	0.53**	168.6
	[0.197–0.827]	[0.301–0.926]	[−618.5–966.7]

Abbreviations: aOR, adjusted odds‐ratio; KES, Kenyan Shillings; OOPE, out‐of‐pocket expenditure.

**p < 0.05.

## 4 DISCUSSION

This study expands our understanding about health insurance and its effects on the probability and the amount spent on medicines by individuals with NCD who report having health insurance compared to those without health insurance. First, we found a stark difference in the characteristics of individuals with and without health insurance. Those with health insurance were wealthier and reported a higher level of formal education which means that NHIF and county specific health insurance enrollment will need to be more targeted towards lower income strata to expand effectively. The monthly fee of KES500 of NHIF is likely to be the most relevant barrier to enrollment for low income households.^27^ Many countries have addressed the problem of low enrollment among low income households by linking insurance enrolment to poverty alleviation program benefits and waiving monthly fees for low income households.^28^ Although the NHIF has implemented a special scheme under NHIF for low income households (Health Insurance Subsidy Program), enrollment barriers for low income households persist.^29^ Recommendations for policy makers in the study counties are more intensive outreach programs and policy legislations that address structural inequities in the enrollment process. Linking enrollment to existing poverty alleviation or social programs may also be effective in boosting enrollment among poorer populations across the counties.

Second, individuals with health insurance had a lower probability of OOP spending on medicines and spent a lower proportion of their health expenditure on medicines which indicates that the insurance had its desired effect in reducing the probability of OOPE.

Third, individuals with health insurance did not spend more OOP on medicines. There is ample literature that shows that scaling up of health insurance can result in an increased spending on auxiliary services and supplies that are not included in the benefit package but necessary to satisfy the needs of the individual.^30^, ^31^ For instance, although many insurance benefit packages provide free ambulatory consultation, they do not include laboratory tests or medicines.^32^ In Kenya, medicines are provided at health facilities free of charge to those with insurance.^33^ However, availability of medicines at the point of care is not guaranteed and stock‐outs have been reported.^34^ which means that individuals need to purchase medicines in the private sector. For insurance administrators and those developing benefit packages, including outpatient medicine benefits is critically important as well as ensuring their availability at the point of service.

## 5 LIMITATIONS

The expenditure on health and medicines were self‐reported which means that recall bias needed to be taken into consideration.^35^ However, the period of recall was 4 weeks which is standard for many expenditure surveys.^35^ Another limitation is that the enrollment in health insurance is largely voluntary; only for those in the formal sector enrollment is mandatory. The data collected does not provide information that can be used to account for the decision to enroll into health insurance or not. The main drivers to enroll are perceived health needs and ability to pay. Since all individuals enrolled in this study had at least one NCD we believe that the individuals experienced a similar need to enroll. Finally, this study is focused on only 8 of the 47 counties in Kenya and therefore cannot be considered nationally representative. Hence, our findings lack generalisability to the entire country.

## 6 CONCLUSION

Kenya has made great strides to scale up UHC including improving access to medicines for NCDs. Prioritising health insurance enrollment of low‐income individuals with NCDs could accelerate access to medicines and financial protection for these needy patients.

## ETHICS STATEMENT

Veronika J. Wirtz reports grants from Access Accelerated, Gilead Sciences, F. Hoffmann‐La Roche, and Amgen outside the submitted work. Peter C. Rockers reports grants from Access Accelerated, Gilead Sciences, F. Hoffmann‐La Roche, and Amgen outside the submitted work. Richard Laing reports non‐financial support from Novartis International AG (if in the last 36 months), Gilead Sciences, and F. Hoffmann‐La Roche outside the submitted work. Edson Servan‐Mori, John Mungai, John Mboya, Monica A. Onyango, Zana Wangari Kiragu does not report conflict of interest.

## Data Availability

The study data set is available on the project website: http://sites.bu.edu/evaluatingaccessnovartisaccess/kenya/data/.
